# Clip or Tattooing: A Comparative Study for Preoperative Colon Cancer Endoscopic Localization

**DOI:** 10.3389/fonc.2022.846900

**Published:** 2022-02-25

**Authors:** Shengyu Zhang, Qiang Wang, Yunlu Feng, Guannan Zhang, Yang Chen, Weiyang Zheng, Xi Wu, Aiming Yang

**Affiliations:** ^1^ Department of Gastroenterology, State Key Laboratory of Complex Severe and Rare Diseases, Peking Union Medical College Hospital, Chinese Academy of Medical Science, Beijing, China; ^2^ Department of General Surgery, State Key Laboratory of Complex Severe and Rare Diseases, Peking Union Medical College Hospital, Chinese Academy of Medical Science, Beijing, China

**Keywords:** preoperative, localization, colon cancer, laparoscopy, endoscopic tattooing

## Abstract

**Background and Aim:**

Preoperative endoscopic markers have been extensively used for the localization of colonic neoplastic lesions in laparoscopic surgery. We conducted this respective cohort study to compare the localization accuracy of two commonly used endoscopic marker strategies (endoscopic clip plus abdominal plain film and endoscopic tattooing).

**Methods:**

Patients who received preoperative colonoscopy localization for colonic neoplasia and underwent an elective laparoscopic operation afterward between 2013 and 2020 were included in this retrospective study. The localization accuracy of the two endoscopic strategies was compared, and the predictors of successful endoscopic localization were identified by multivariate regression.

**Results:**

In total, 195 patients [average age 62.4 ± 9.2 years, 123 male (63.1%)] undergoing preoperative colonoscopy localization and subsequent laparoscopic colectomy for colonic neoplasms were included. Endoscopic localization was finally proven to be successful in 150 (76.9%) patients in the surgery. Compared to the tattooing group, patients who had successful localization for colonic lesions were fewer in the clip group (64 of 101 cases, 63.4% vs. 86 of 94 cases, 91.5%, *p* < 0.001). The multivariate regression analysis showed that the endoscopic tattooing strategy, endoscopic clip strategy, and lesion location were all predictors for successful localization (all with *p* < 0.001).

**Conclusion:**

Compared with endoscopic clip plus abdominal plain film, endoscopic tattooing had higher localization accuracy and less intraoperative colonoscopy counseling; the endoscopic clip strategy, tattooing strategy, and colonic lesion location were all predictors of successful endoscopic localization.

## Introduction

Colon cancer is evolving worldwide, ranking third in terms of incidence and second in terms of mortality (1). Colonoscopy is an essential tool not only for colon cancer screening and diagnosis but also for surgical procedure planning. Due to its minimal invasiveness, similar safety, and identical long-term results as compared with conventional open surgery, laparoscopic surgery has been widely accepted as one of the standard treatments for colon cancer (2).

Due to the lack of tactile sensation, intraoperative identification of neoplastic lesions may be difficult, particularly with smaller lesions (3). A simple and effective strategy for accurate localization of primary lesions during laparoscopic procedures is essential for safe segmental colon resection and appropriate surgical cutting margins.

Estimating the location of colon tumors with or without marking as a guide for resection has been extensively used with variable accuracy in precise anatomical identification of the primary lesion. Conventional colonoscopy localization depends on many factors, including endoscopist expertise, tumor location, cecal intubation, bowel preparation, and tumor obstruction; and incorrect localization was reported in the meta-analysis to be 15.4% (95% CI, 12.0–18.7) (4). Routine CT scans lead to a much higher inaccuracy rate than colonoscopy, especially for small tumors (5).

To improve the localization accuracy, endoscopic markers are applied. Endoscopic clip placement combined with abdominal radiography is reported to be a cost-effective and plausible strategy with high accuracy, in which high-density shadows of the clips attached near the colon lesion can be easily recognized on supine abdominal radiographs (6, 7). As an important strategy for localizing colon lesions for subsequent endoscopic treatment, endoscopic tattooing is also used in colon tumor localization for laparoscopic surgery but has been found to have a pooled incidence of localization errors of 9.5% (95% CI, 5.7–13.3) (8). However, there has not been a comparative study comparing two endoscopic localization strategies for the intraoperative estimation of colonic lesions of interest.

Therefore, we conducted this retrospective cohort study to compare the localization accuracy of endoscopic clips plus abdominal plain film and endoscopic tattooing and to identify the clinical factors that could predict successful localization.

## Patients and Methods

**Figure 1:**
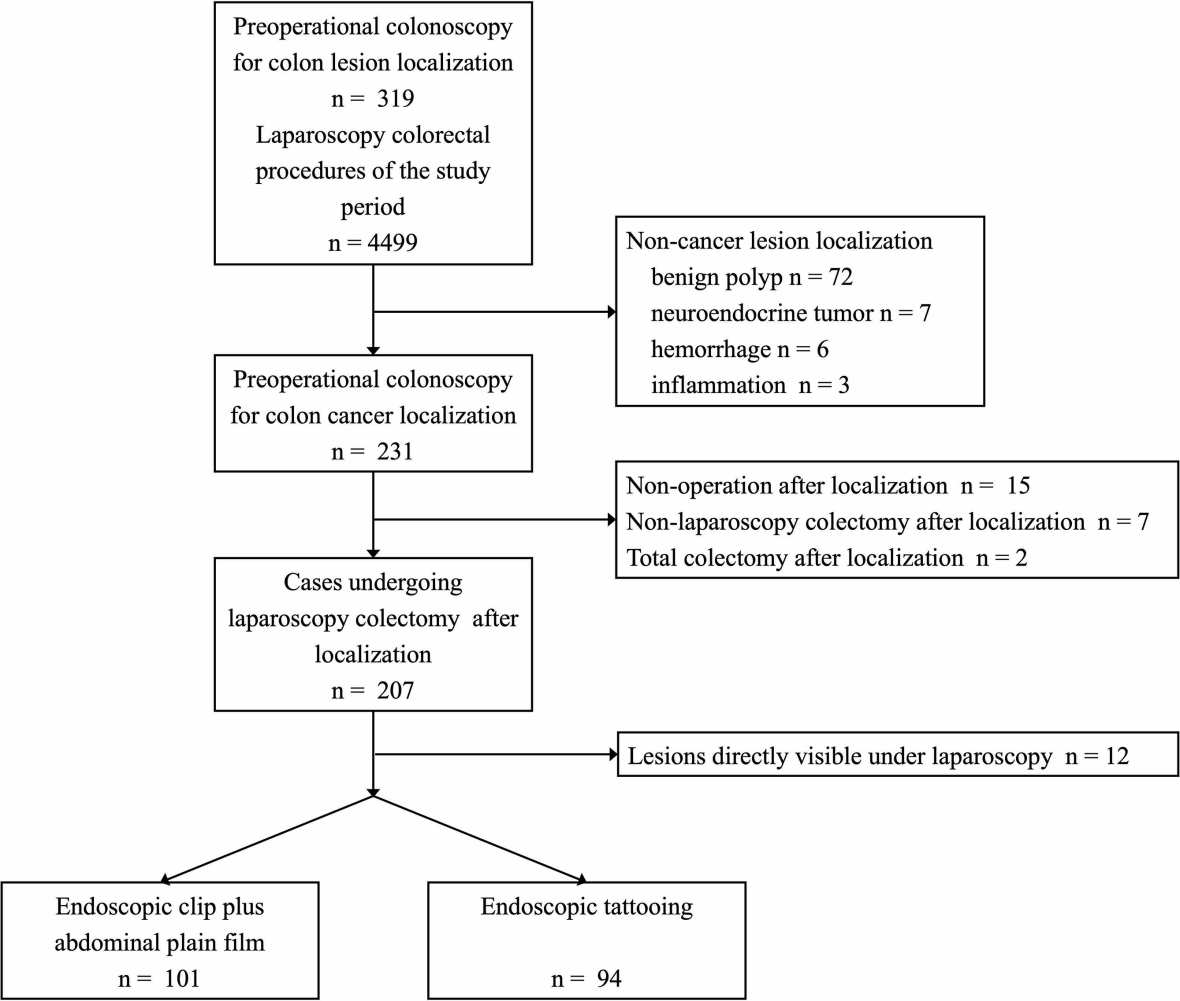
The flowchart of patient inclusion.

Patients who received preoperative colonoscopy localization for colonic neoplasia and underwent an elective laparoscopic operation afterward in a tertiary hospital (Peking Union Medical College Hospital (PUMCH)) between 2013 and 2020 were retrospectively reviewed and included. This retrospective study was approved by the Institutional Review Board in PUMCH. The flowchart of patient inclusion is shown in **Figure 1**.

The demographic and clinical information (age, sex, body mass index, previous abdominal/pelvic surgery history, surgery note, and pathology information) were collected from the original medical record; the CT and abdominal plain film images and reports were obtained from the health information system in PUMCH; colonoscopy and localization reports were obtained from the dataset in the gastrointestinal endoscopy center.

### Colonoscopy Information

Sites of the primary lesions were divided into the right hemicolon (cecum to hepatic flexure), transverse colon, descending colon (splenic flexure and descending colon), sigmoid colon (descending-sigmoid junction and sigmoid colon), and rectum (rectosigmoid junction and rectum) based on the surgery notes and endoscopic reports and further classified as the proximal colon (cecum to splenic flexure, excluding splenic flexure) and the distal colon (splenic flexure to rectum). In cases of multiple preoperative colonoscopies, only the one prior to surgical resection with endoscopic localization was used as a reference in each patient; in cases where more than one lesion was identified during colonoscopy, only the lesion that required surgical resection was analyzed as a reference.

**Figure 2:**
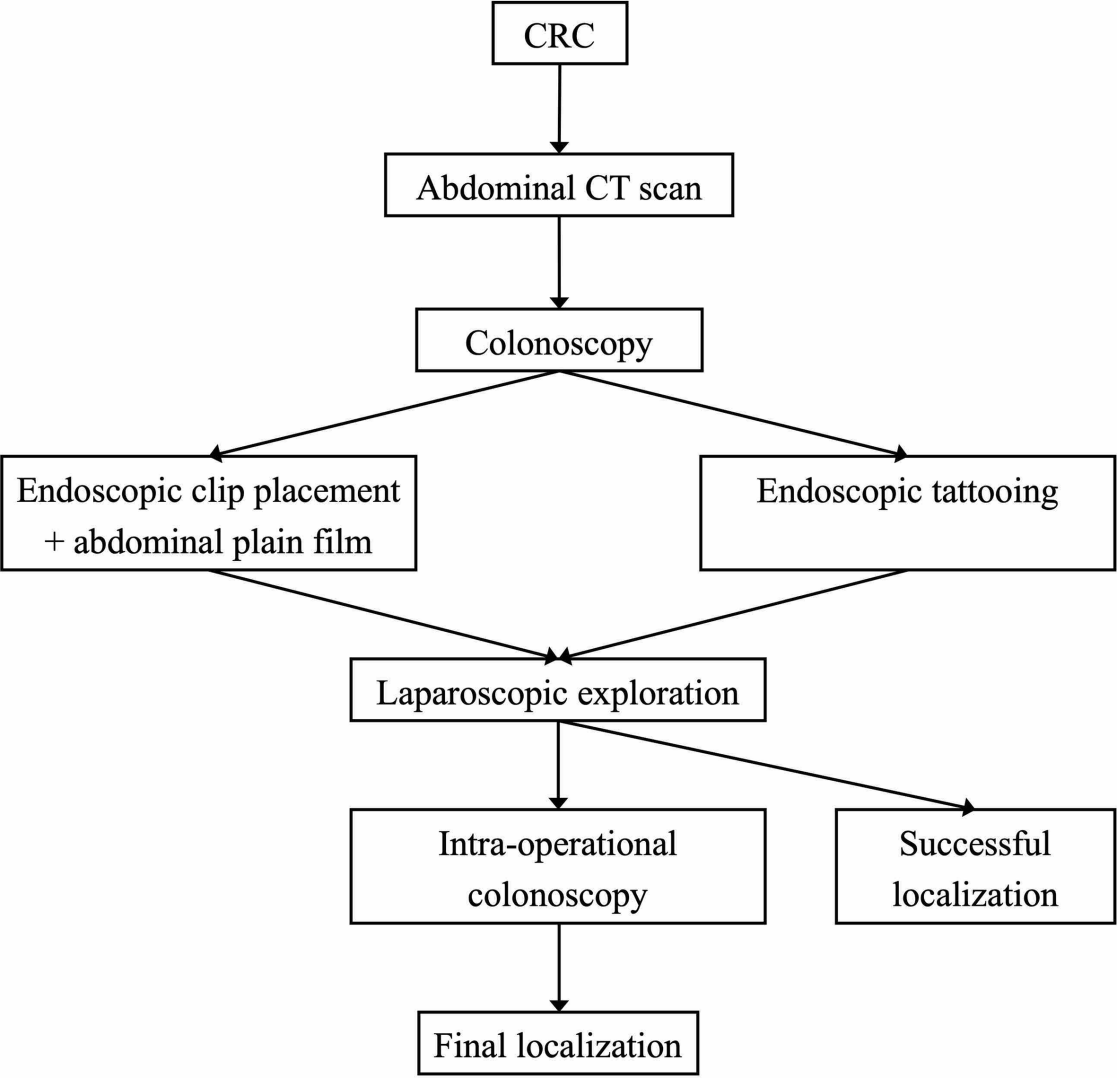
Two endoscopic localization strategies for colonic tumors in laparoscopic surgery.

Most patients received CT first and then the endoscopic procedure. The preoperative colonoscopy localization followed two different strategies: the clip plus X-ray strategy, in which the endoscopist would place through-the-scope clip(s) (Long clip, Olympus^®^; Tokyo, Japan) near the colonic tumor, and a supine abdominal plain film would be ordered immediately after the endoscopy procedure to localize the lesion (6) (the abdominal film was personally interpreted by experienced radiologists); and the tattooing strategy, in which endoscopists would tattoo the lesions with nanocarbons in a standardized way (9) to make the tumor visible under laparoscopy. The decision for either strategy or its specific technical details was left at the discretion of the endoscopists, and all patients received laparoscopic procedures on the next day of the colonoscopy. The endoscopic localization protocol is shown in **Figure 2**.

### Surgery Information

The intraoperative location in the surgery note was considered to be the final location of lesions, and the concordance rates of CT and colonoscopy with surgery were also calculated. The discrepancies in tumor location between the colonoscopy and surgery were recorded, regardless of whether it could lead to a change in the original surgical plan. Successful localization meant that the surgeons could visualize the endoscopic markers and/or complete the colectomy without the intraoperative colonoscopy; colonic lesions directly visible under laparoscopic exploration (for high T staging) were excluded from the final analysis (**Figure 1**). The decision for intraoperative colonoscopy was left at the discretion of the surgeons.

Informed consent was obtained for the planned surgical procedure prior to the surgery; the surgery plan change referred to a procedure different from the planned one that was undertaken during the surgery. Extending the resection area was defined as the inclusion of one or more colonic segments within the resected specimen compared to the planned procedure.

The surgery note was also checked for operation time and blood loss volume. The pathology reports were reviewed for R_0_ resection, the length of the surgical specimen, and the tumor staging.

### Statistical Analysis

Patients were compared according to the endoscopic localization strategy and successful localization. Univariate analysis was performed to identify factors associated with successful localization. Categorical variables are presented as numbers (%), and continuous variables are presented as the mean ± SD or median and interquartile range (IQR), depending on the distribution. Categorical variables were compared using χ^2^ and Fisher’s exact tests, when appropriate, whereas for numerical variables, Student’s t-test and Mann–Whitney test were used depending on the distribution. Multivariate analysis using binary logistic regression included variables identified to be significant (*p* ≤ 0.10) in the univariate analysis. Statistical analysis was accomplished in SPSS (IBM, NY, USA; version 23.0). A two-tailed *p*-value of less than 0.05 was considered to be statistically significant.

## Results

In total, 195 patients [average age 62.4 ± 9.2 years, 123 male (63.1%)] undergoing preoperative colonoscopy localization and subsequent colectomy for colonic neoplasms were included in this study. All lesions were malignant (T1, 12.8%; T2, 32.8%; T3, 46.7%; and T4, 7.7%), and all cases had R_0_ resection according to the final pathological reports. Preoperative CT was accomplished in 168 (86.2%) patients, which showed colonic lesions in 109 (55.9%) cases; CT localization was concordant with surgery findings in 86 (44.1%) cases. Colonoscopy was finished in all cases preoperatively, and major patients (153 cases, 78.5%) had well-prepared bowels. Endoscopic localization was achieved in all cases and finally proven to be “successful” in 150 (76.9%) patients in the surgery, and intraoperative colonoscopy was essential in 45 (23.1%) cases.

### Clip Versus Tattooing: Univariate Analysis

There were 101 (51.8%) patients receiving endoscopic clips and 94 (48.2%) patients receiving endoscopic tattooing (**Table 1**). The clips were more frequently placed “cranially and caudally” (31 cases, 30.7%) and “*in situ* (just beside the tumor lesion)” (50 cases, 49.5%), while nanocarbon tattooing was more frequently injected “caudally” (59 cases, 62.8%); the average number of endoscopic clips was more than that of endoscopic tattooing (2 IQR [2, 3] vs. 1 [1, 1], *p* < 0.001). Otherwise, compared to the tattooing group, patients who had successful localization for colonic lesions were significantly less in the clip group (64 cases, 63.4% vs. 86 cases, 91.5%, *p* < 0.001), so intraoperative colonoscopy was more frequently practiced in the clip group accordingly (37 cases, 36.6% vs. 8 cases, 8.5%, *p* < 0.001). In addition, there were significantly more patients who had cecal intubation during colonoscopy in the clip group (58 cases, 57.4% vs. 39 cases, 41.5%, *p* = 0.032), but this did not increase the concordant localization rate of colonoscopy. There were no major differences in colonic lesion location, surgery plan change, operation time, or surgical specimen length between the two groups. The blood loss volume in most surgery notes was recorded as “minimal” or “less than 20 ml,” so it was not analyzed.

**Table 1:** The demographic and clinical characteristics of the study patients in the endoscopic clip and tattooing groups.

	Overall (n = 195)	Endoscopic clip (n = 101)	Endoscopic tattooing (n = 94)	*p*-Value
Age, years	62.4 ± 9.2	61.8 ± 9.8	63.1 ± 8.6	0.331
Male, n (%)	123 (63.1)	61 (60.4)	62 (66.0)	0.421
BMI, kg/m^2^	24.8 ± 3.6	24.5 ± 2.4	24.9 ± 3.9	0.742
Previous abdominal or pelvic surgery, n (%)	40 (20.5)	18 (17.8)	22 (23.4)	0.335
Intact colon, n (%)	195 (100)	101 (100)	94 (100)	1.000
Preoperative CT, n (%)	168 (86.2)	88 (87.1)	80 (85.1)	0.836
Lesion seen on CT, n (%)	109 (55.9)	60 (59.4)	49 (52.1)	0.334
CT localization concordance, n (%)	86 (44.1)	47 (46.5)	39 (41.5)	0.478
**Colonoscopy**				
Cecal intubation, n (%)	97 (49.7)	58 (57.4)	39 (41.5)	0.032
Bowel preparation				0.070
Good, n (%)	153 (78.5)	73 (72.3)	80 (85.1)	
Poor, n (%)	42 (21.5)	28 (27.7)	14 (14.9)	
Distance from the anus to lesion, cm^‡^	22 [14, 35]	23 [13, 40]	20 [15, 30]	0.504
Colonoscopy localization concordance, n (%)	167 (85.6)	87 (86.1)	80 (85.1)	0.837
**Endoscopic markers**				
Marker position				<0.001
Cranial, n (%)	11 (5.6)	2 (2.0)	9 (9.6)	
Caudal, n (%)	77 (39.5)	18 (17.8)	59 (62.8)	
Cranial + caudal, n (%)	32 (16.4)	31 (30.7)	1 (1.0)	
* In situ*, n (%)	75 (38.5)	50 (49.5)	25 (26.6)	
Number of markers^‡^	1 [1, 2]	2 [2, 3]	1 [1, 1]	<0.001
**Laparoscopic operation**				
Successful localization, n (%)	150 (76.9)	64 (63.4)	86 (91.5)	<0.001
Intraoperative colonoscopy, n (%)	45 (23.1)	37 (27.7)	8 (5.3)	<0.001
Lesion location after surgery^†^				0.608
Right colon, n (%)	37 (19.0)	21 (20.8)	16 (17.0)	
Transverse colon, n (%)	17 (8.7)	8 (7.9)	9 (9.6)	
Descending colon, n (%)	16 (8.2)	11 (10.9)	5 (5.3)	
Sigmoid colon, n (%)	67 (34.4)	33 (32.7)	34 (36.2)	
Rectum, n (%)	58 (29.7)	28 (27.7)	30 (31.9)	
Operation time, h	2.30 ± 0.88	2.41 ± 0.83	2.20 ± 0.92	0.115
Surgical specimen length, cm	15.5 ± 5.6	14.2 ± 4.9	15.5 ± 5.9	0.178

Note. Localization concordance: coincidence with final location during surgery. Bowel preparation: good, equivalent to the Boston bowel preparation scale of more than 6 points; poor, equivalent to less than 6 points. Marker position: cranial, located within 3 cm cranially to the lesion; caudal, located within 3 cm caudally to the lesion; in situ, located just beside the lesion. Tumor visualization: tumors can be directly visualized under laparoscopy. Lesion location: right colon, includes cecum, ascending colon, and hepatic flexure; transverse colon, excludes hepatic and splenic flexure; descending colon, includes splenic flexure and descending colon; sigmoid colon, includes the descending-sigmoid junction and sigmoid colon; rectum, includes the rectosigmoid junction and rectum.

BMI, body mass index.

^†^Fisher’s exact test.

^‡^The Mann–Whitney test.

### Successful Versus Unsuccessful Localization: Univariate Analysis

There were 150 (76.9%) cases whose endoscopic localization was successful, while 45 (23.1%) cases received unsuccessful localization (**Table 2**). Compared to the unsuccessful group, there were significantly more patients receiving endoscopic tattooing (86 cases, 57.3% vs. 8 cases, 17.8%, *p* < 0.001), and the opposite was true for the endoscopic clip (64 cases, 42.7% vs. 37 cases, 82.2%, *p* < 0.001). There were more patients in the successful group with lesions located in the proximal colon (52 cases, 34.7% vs. 2 cases, 4.4%, *p* < 0.001) and fewer patients with lesions in the distal colon (98 cases, 65.3% vs. 43 cases, 95.6%, *p* < 0.001) than in the unsuccessful group. In addition, the operation time in the successful group was comparable to that in the unsuccessful group.

**Table 2:** The demographic and clinical characteristics of the study patients in the endoscopic localization successful/unsuccessful group.

	Overall (n = 195)	Successful cases (n = 150)	Unsuccessful cases (n = 45)	*p*-Value
Age, years.	62.4 ± 9.2	63.0 ± 9.1	60.4 ± 9.2	0.099
Male, n (%)	123 (63.1)	93 (62.0)	30 (66.7)	0.569
BMI, kg/m^2^	24.8 ± 3.6	24.8 ± 3.4	25.0 ± 4.7	0.907
Previous abdominal or pelvic surgery, n (%)	40 (20.5)	32 (21.3)	8 (17.8)	0.604
Preoperative CT, n (%)	168 (86.2)	130 (86.7)	38 (84.4)	0.705
Lesion seen on CT, n (%)	109 (55.9)	86 (57.3)	23 (51.1)	0.461
**Colonoscopy**				
Cecal intubation, n (%)	97 (49.7)	76 (50.7)	21 (46.7)	0.638
Bowel preparation				0.775
Good, n (%)	153 (78.5)	117 (78.0)	36 (80.0)	
Poor, n (%)	42 (21.5)	33 (22.0)	9 (20.0)	
Endoscopic clip, n (%)	101 (51.8)	64 (42.7)	37 (82.2)	<0.001
Endoscopic tattooing, n (%)	94 (48.2)	86 (57.3)	8 (17.8)	<0.001
**Laparoscopic operation**				
Lesion location after surgery				<0.001
Proximal colon, n (%)	54 (27.7)	52 (34.7)	2 (4.4)	
Distal colon, n (%)	141 (72.3)	98 (65.3)	43 (95.6)	
Operation time, h	2.30 ± 0.88	2.28 ± 0.90	2.40 ± 0.81	0.445
Surgical specimen length, cm	15.5 ± 5.6	13.4 ± 5.1	15.4 ± 5.6	0.114

Bowel reparation: good, equivalent to the Boston bowel preparation scale of more than 6 points; poor, equivalent to less than 6 points. Lesion location: proximal colon, including cecum, ascending colon, hepatic flexure, and transverse colon, excluding splenic flexure; distal colon, including splenic flexure, descending colon, sigmoid colon, and rectum.

BMI, body mass index.

### Multivariate Regression Analysis for Successful Localization

The multivariate regression analysis (binary logistic regression; step forward method, likelihood ratio) showed that endoscopic tattooing, endoscopic clip, and lesion location were all predictors for successful localization for colonic neoplastic lesions (all with *p* < 0.001) (**Table 3**).

**Table 3:** Logistic regression for successful localization of colon cancer.

Effect	Odds ratio (95% CI)	*p*-Value
Endoscopic tattooing	114.8 (22.8, 579.0)	<0.001
Endoscopic clip	15.3 (3.7, 63.7)	<0.001
Lesion location		
Proximal colon	1.00 (REF)	
Distal colon	0.068 (0.015, 0.303)	<0.001

Lesion location: proximal colon, including cecum, ascending colon, hepatic flexure, and transverse colon, excluding splenic flexure; distal colon, including splenic flexure, descending colon, sigmoid colon, and rectum.

### Discrepancies Between Endoscopic and Surgical Localization

There were discrepancies between endoscopic and surgical localization of the colonic lesions in 18 cases [6 cases (5.9%) in the clip group vs. 12 cases (12.8%) in the tattooing group, *p* = 0.100], leading to a change in the final surgical procedure in 10 cases (**Table 4**). Among the patients whose procedures were changed, more than half of the cases (6 cases, 60%) underwent a completely different segmental resection from what was initially planned; in 2 cases, a more extensive resection was required than originally planned; 2 cases switched to open surgery were due to extensive intraperitoneal adhesion.

**Table 4:** The incorrectly localized lesions and changes in the surgery.

Incorrect location	n (%)	Actual location	n	Surgery plan change	n
Transverse colon	3 (16.7)	Ascending colon	3	Switch to the open surgery^*^	1
Descending colon	3 (16.7)	Transverse colon	1	Switch to the open surgery^*^	1
		Sigmoid colon	2	/	
Sigmoid colon	12 (66.7)	Rectum	12	Resection of other segments of the colon	6
				Extend the resection area	2

Lesion location: transverse colon, excluding hepatic and splenic flexure; sigmoid colon, including the descending-sigmoid colon junction and sigmoid colon; rectum, including the rectosigmoid junction and rectum.

^*^Cases with laparoscopy switched to open surgery were due to extensive intraperitoneal adhesion.

## Discussion

This was a retrospective study that compared the localization accuracy rate of endoscopic clip plus abdominal radiology and endoscopic tattooing and tried to find the independent predictors of successful endoscopic localization.

**Figure 3:**
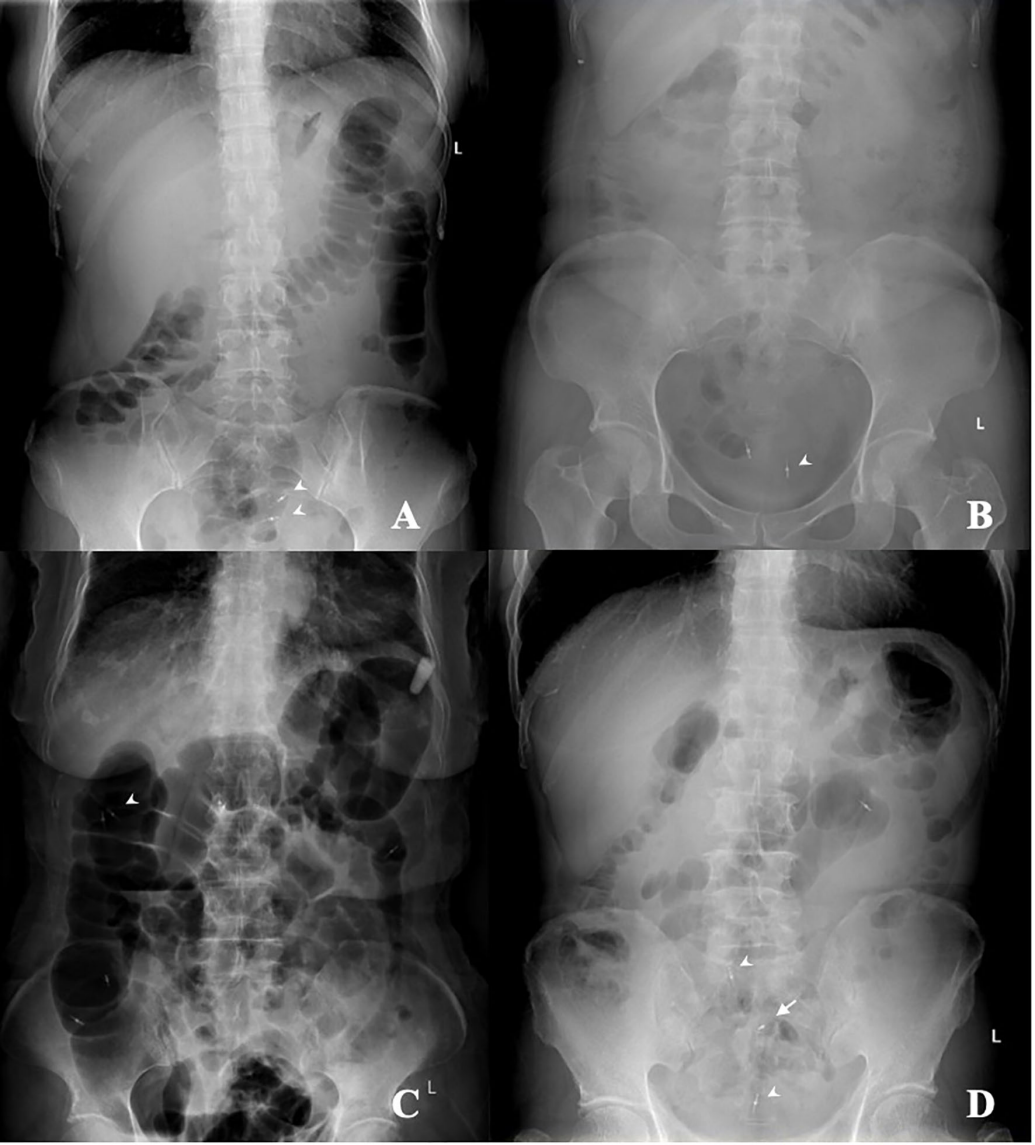
The supine abdominal plain film after clip placement for tumor localization. **(A)** The appropriately air-inflated colon and the clips (arrowhead) near the tumor in the upper rectum. **(B)** The poorly air-inflated colon and the clips (arrowhead) near the tumor in the rectosigmoid junction. **(C)** The over air-inflated colon and the overlapped lumen of the ascending colon and transverse colon, and the clips (arrowhead) failed to localize the tumor in the transverse colon. **(D)** Several clips in the sigmoid colon, with two clips (arrowhead) placed after polyp removal and one clip (arrow) placed near the tumor, which would create confusion.

In this study, we demonstrated that the localization accuracy rate of the tattooing group was significantly higher than that of the clip group (91.5% vs. 63.4%, *p* < 0.001), and both endoscopic clips (odds ratio (OR) = 15.3, 95% CI, 3.7–63.7, *p* < 0.001) and tattooing (OR = 114.8, 95% CI, 22.8–579.0, *p* < 0.001) were predictors of endoscopic localization success, which implied that both localizing strategies were effective but tattooing was possibly superior. Cai et al. reported endoscopic clip placement followed by immediate supine abdominal radiograph in the “unreliable” group (tumors that were not in the range from the cecum to the hepatic flexure), with high localizing accuracy (113 cases correctly localized in 131 cases, 86.3%). However, we did not find such a high success rate for endoscopic clip plus supine abdominal plain film, although the number of clips placed in our study was comparable with that of Cai et al. The localization failure seemed to relate to the detachment of the metal clips before radiography, the position change and overlap of different segments of the colon, and the interference from other clips placed after polyp removal (**Figure 3**). Therefore, we suggest the placement of multiple clips in the normal tissue near the neoplastic lesions and immediate radiological examination (<30 min) in case the clip falls off (6), appropriate air inflation to avoid overexpansion and overlap of the colonic lumen, and no clip placement other than localizing goals unless necessary; all these technical details may improve the success rate of localization. There are also other methods of finding endoscopic clips during laparoscopic procedures, such as laparoscopic ultrasound to detect metal clips (10) and full-color fluorescent laparoscopy to find fluorescent clips (11), which are not only highly dependent on the user and device but also time-consuming.

**Figure 4:**
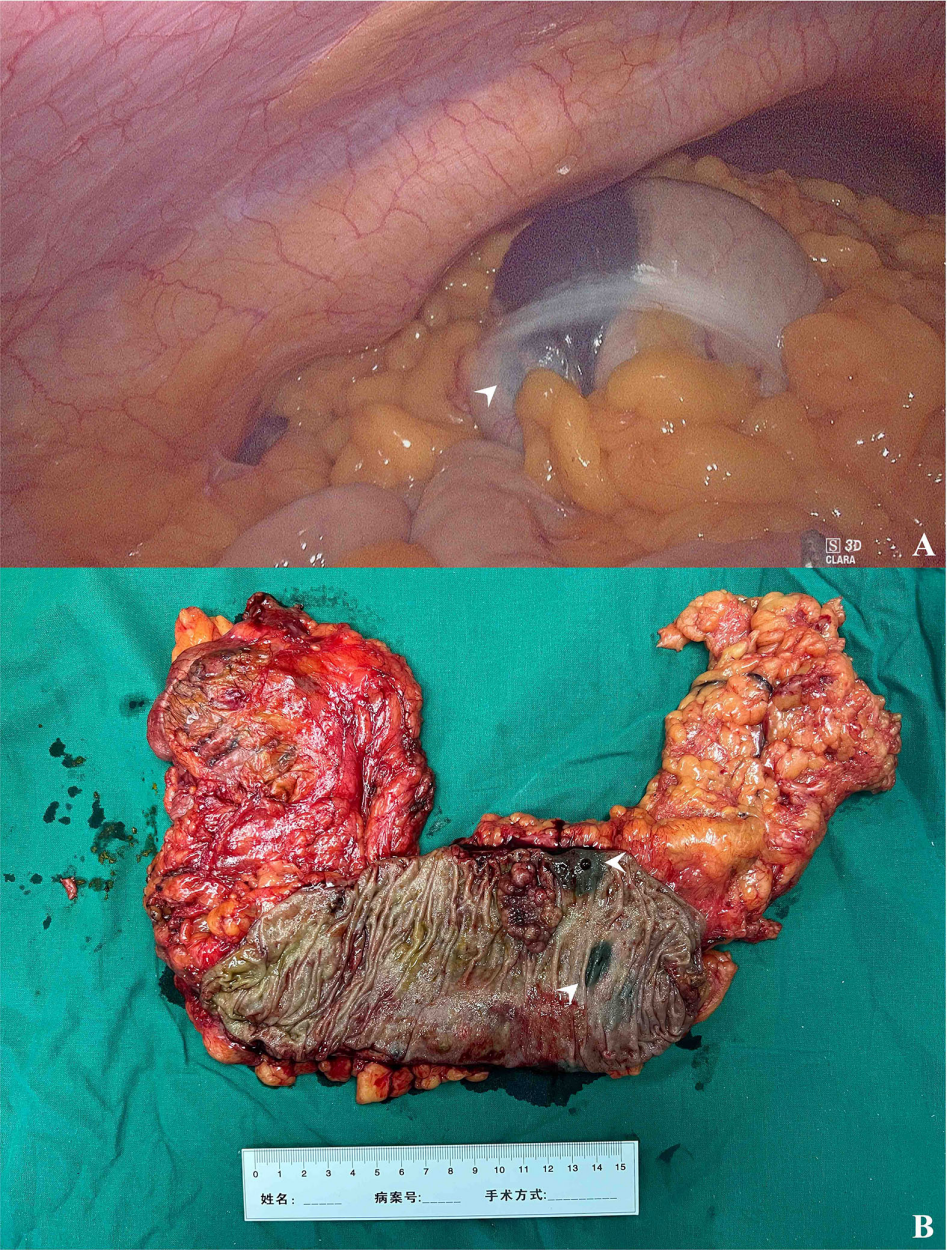
The intraoperative finding of tattooed colon cancer. **(A)** The black patch in the hepatic flexure under laparoscopy (arrowhead). **(B)** The resected surgical specimen and two tattoos caudally to the cancer lesion (arrowheads).

On the other hand, endoscopic tattooing is usually used for colonic lesion localization (12, 13). In published studies, the localization accuracy varied from 70% to 97.9% (14–17), and the pooled localization accuracy was 90.5% in a meta-analysis (8), which was consistent with our findings. The unsuccessful tattooing localizations all occurred in the lesions in the distal colon (1 case in the descending colon, 10 cases in the sigmoid colon, and 1 case in the superior part of rectum), possibly because of injection at the site of the mesenteric colon or insufficient injection of nanocarbon, which would make the lesions invisible under the laparoscope. Therefore, although one tattoo seemed to be enough to identify lesions in a large number of cases in this study (91.5%), we suggest the placement of the tattoos circumferentially at two or three sites to avoid mesenteric colon injection and facilitate laparoscopic identification following international agreement (**Figure 4**) (13). It was also notable that 30 patients (31.3%) in the tattoo group had rectal cancer, most of which were located in the rectosigmoid junction and upper rectum (the mean distance from the anus to the lesion was 12.9 ± 5.3 cm), instead of the middle and lower rectum where tattooing could color the whole mesorectal plane and therefore make the surgical procedure within the right plane more difficult (13).

It is well known that endoscopic tattooing can cause some unusual complications, such as fat necrosis, adhesion formation, perforation, peritonitis, and even liver abscess (6, 18, 19). In this study, we placed the nanocarbon tattoo in a standardized saline bleb technique to reduce the risk of peritoneal spillage and deep muscular injury (9) with no tattooing-associated complications occurring. The tattoos were mostly injected caudally or just beside the lesions in this study (in total 84 cases, 89.4%), and technically the location of the tattoo would not influence the localization result if clearly documented in the endoscopy report. Considering the possibility of lumen occlusion by the neoplastic lesion and the potential risk of tumor implantation through the transmural needle tract (20), placement of the tattoo 3–5 cm caudally to the lesion seemed practical (13).

In addition, we demonstrated that lesion location (distal colon, OR = 0.068, 95% CI, 0.015–0.303, *p* < 0.001) was an independent predictor for successful localization; in this study, discrepancies between preoperative endoscopic and intraoperative localization all occurred in the distal colon. Previous studies also reported similar findings: Fernandez et al. found that patients with transverse or distal lesions were more likely to have a change in final surgical management compared to proximal lesions (29.7% vs. 3.9%, *p* < 0.001) (17); Luis et al. found that major discrepancies between endoscopic reports and surgery findings were significantly higher in the left colon (17/185, 9.1%) than in the right colon (3/160, 1.9%; *p* = 0.045) (21); Saleh et al. also reported the left-side colonic lesions to be a risk factor for inaccurate tumor location (*p* = 0.012) (22). The reason for this might be that the left-sided anatomical landmarks (splenic flexure and rectosigmoid junction) are sometimes difficult to recognize; the redundant sigmoid colon lumen could change position easily and overlap with that of the descending colon, which complicates endoscopic clip localization *via* radiography; and tattoo injection in the mesenteric part of the descending colon may make the nanocarbon tattoos invisible.

The localization discrepancies led to a change in the initial surgical plan in 8 cases (including completely different segmental resection and extended resection areas), all of which occurred in the sigmoid colon and rectum. From a surgical viewpoint, compared to right colon lesions, which are essentially treated with right hemicolectomy, lesions in the left colon involve different methods of resection, such as left colectomy, sigmoid colectomy, and anterior resections, depending on the precise localization preoperatively in the key areas (23).

Therefore, the endoscopic tattooing strategy probably seemed better than the endoscopic clip plus X-ray strategy due to its simplicity, accuracy, and direct visuality in laparoscopy. However, the tattoos could not indicate the position for the first trocar (served as camera port) preoperatively, especially for tumors of the transverse colon. Additionally, considering the variability and inconsistencies in endoscopic tattooing practice (12, 24), a standardized protocol should be implemented and followed in each endoscopy center and should be made clear to all members of the tumor multidisciplinary committee, while details of the tattoo injection should be both stated and photodocumented in the report clearly (13).

This study has limitations. First, our study was limited by its retrospective design and single-institution experience, which may limit the generalizability of our findings. The decision to place a tattoo or clip to localize the lesion was at the discretion of the endoscopists, and colonic tumors were explored and resected by different surgeons, which introduced potential selection bias. Additionally, we could not demonstrate the time between the endoscopic procedure and abdominal plain film, possibly because of clip detachment and unsuccessful localization; the time spent locating the colon tumor during laparoscopy was not recorded in the surgery note, so we could only compare the total time of operation, which was shown to be similar between successful and unsuccessful localized cases. Second, only a limited percentage of patients (7.1%) undergoing laparoscopy colorectal procedures received preoperative endoscopic localization in our hospital, while others would need intraoperative colonoscopy or change to open surgery in case of unsuccessful laparoscopic localization. We still have a lot to do with the generalization of colon lesion endoscopic localization. Third, there is no standardized protocol for the placement of endoscopic markers in our center, so the position and number of markers varied in this study. Fourth, we did not consider the economic cost or difficulty of endoscopic procedure for either strategy. Finally, to demonstrate the possible superiority of endoscopic tattooing, randomized prospective studies for optimizing the tattoo injection plan (site, number, and nanocarbon volume) should be conducted to increase the accuracy and decrease complications of this procedure.

In conclusion, compared with the endoscopic clip plus abdominal plain film, the endoscopic tattooing strategy had higher localizing accuracy and less intraoperative colonoscopy counseling; the endoscopic clip strategy, tattooing strategy, and colonic lesion location are all predictors of endoscopic localization success.

## Data Availability Statement

The original contributions presented in the study are included in the article/supplementary material. Further inquiries can be directed to the corresponding authors.

## Ethics Statement

The studies involving human participants were reviewed and approved by the Institutional Review Board of Peking Union Medical College Hospital. The ethics committee waived the requirement of written informed consent for participation.

## Author Contributions

SZ: study design, endoscopist performing the procedure, statistical analysis, interpretation of data, and drafting of the manuscript. QW: endoscopist performing the procedure, statistical analysis, interpretation of data, and critical revision of the manuscript. YF: endoscopist performing the procedure, interpreting the data, and critically revising the manuscript. YC: endoscopist performing the procedure and critical revision of the manuscript. WZ: endoscopist performing the procedure. XW: study concept and critical revision of the manuscript. AY: study concept, critical revision, and final approval of the manuscript. All authors listed have made a substantial, direct, and intellectual contribution to the work and approved it for publication.

## Funding

This study was supported by the Beijing Science and Technology Program (Z181100001618013) and Peking Union Medical College Education Reform Program (2019zlgc1006).

## Conflict of Interest

The authors declare that the research was conducted in the absence of any commercial or financial relationships that could be construed as a potential conflict of interest.

## Publisher’s Note

All claims expressed in this article are solely those of the authors and do not necessarily represent those of their affiliated organizations, or those of the publisher, the editors and the reviewers. Any product that may be evaluated in this article, or claim that may be made by its manufacturer, is not guaranteed or endorsed by the publisher.
